# Incidence and Levels of Deoxynivalenol, Fumonisins and Zearalenone Contaminants in Animal Feeds Used in Korea in 2012

**DOI:** 10.3390/toxins6010020

**Published:** 2013-12-23

**Authors:** Dong-Ho Kim, In-Hye Lee, Woo-Hyun Do, Woo-Seon Nam, Hua Li, Han-Sub Jang, Chan Lee

**Affiliations:** 1 National Agricultural Products Quality Management Service, Seoul 150-804, Korea; E-Mail: anoldmu@korea.kr; 2 School of Food Science and Technology, College of Natural Science, Chung-Ang University, Anseong-Si 456-756, Korea; E-Mails: dkr1313@naver.com (I.-H.L.); dwh717@nate.com (W.-H.D.); nws0326@naver.com (W.-S.N.); lihua8272@nate.com (H.L.); jjhs@korea.kr (H.-S.J.)

**Keywords:** *Fusarium*, deoxynivalenol, fumonisin B_1_, fumonisin B_2_, zearalenone, compound feeds, feed ingredients

## Abstract

The objective of this study was to evaluate the occurrence and levels of deoxynivalenol (DON), fumonisins B_1_ and B_2_ (FBs), and zearalenone (ZEN) contaminants in animal feeds used in Korea in 2012. Contamination with DON was observed in 91.33% and 53.33% in compound feeds and feed ingredients, respectively. Among compound feeds, poultry layer feed (laying) exhibited the highest contaminant level of 1.492 mg/kg. FBs contaminants were present in compound feeds and feed ingredients at 93.33% and 83.33%, respectively. Most poultry broiler (early) feeds were highly contaminated with FBs, and one of these feeds detected the level as 12.823 mg/kg as the highest level. The levels of ZEN in compound feeds and feed ingredients were 71.33% and 47%, respectively. Ninety-eight percent of compound feeds for cattle were contaminated with ZEN, and the highest contamination level of 0.405 mg/kg was observed in cattle fatting feeds.

## 1. Introduction

Members of the mycotoxin-producing fungal species, *Fusarium*, occur as facultative saprophytes and parasites. They are widespread in nature, and frequently contaminate many crops (corn, wheat, rye, oats, and rice) by producing a wide range of toxins [1]. The most hazardous *Fusarium* mycotoxins, in terms of animal health and productivity, are deoxynivalenol (DON), zearalenone (ZEN), fumonisins (FBs) and moniliformin [2].

DON, which is produced by *F. graminearum* [3] and *F. culmorum* [4], is one of the most common contaminants of corn, wheat, and barley worldwide. DON was characterized as a trichothecene and was given the name “vomitoxin” because of its emetic effect on swine [5,6]. FBs, which are diesters of propane-1,2,3-tricarboylic acid and various 2-amino-12,16-dimetylpolyhydroxyeicosanes, are natural contaminants in maize and maize-based feeds and foods worldwide. FBs such as FB_1_, FB_2_, and FB_3_ are particularly produced by *F. verticillioides*, and the related *F. proliferatum* [7]. FBs are commonly detected in foodstuffs such as corn, sorghum, asparagus, rice, beer, and beans. In long-term feeding studies, purified FB_1_ caused liver and kidney tumors in rodents [8]. ZEN is one of the most widely distributed mycotoxins produced by the *Fusarium* species *F. graminearum* and *F. culmorum*. It is found worldwide in various cereals such as maize, barley, wheat, and sorghum, and is classified as an estrogenic mycotoxin because it frequently uses hyperestrogenic syndrome in animals. ZEN-contaminated feed or grain, when consumed by livestock, can contribute to a wide variety of reproductive problems.

The presence of mycotoxins in grains and animal feeds has been reported worldwide for decades. Mycotoxin contamination of feed has become a major concern in animal feed science because it can cause acute or chronic mycotoxicosis in animals [9]. For these reasons, the regulation of mycotoxin levels in feed is of great interest, and the commission regulation of the European community set maximum levels (MLs) for FBs, DON, and ZEN in various animal feeds in 2007. Some countries in Asia, for example China and Japan, also have guidelines for these toxins in feeds. The MLs for FBs for complete feed in China was set to 0.500 mg/kg, 0.500 mg/kg for DON, and 0.100 mg/kg for ZEN. However, there are still no guideline values for these toxins in animal feed in Korea. This study therefore aimed to assess the occurrence and levels of DON, FBs, and ZEN in animal feeds distributed in South Korea in 2012, to better understand the contamination levels of mycotoxins in feeds.

## 2. Results and Discussion

### Contamination Levels

#### Contamination Levels of DON in Compound Feeds and Feed Ingredients

As shown in Table 1, 100% of the cattle feed samples were contaminated with DON, with the levels of contamination ranging from 0.131 to 1.000 mg/kg. In swine feed, 44 out of 50 samples (88%) were contaminated with DON, with levels of 0.037–0.982 mg/kg, and a mean contamination level of 0.297 mg/kg. The fatting growing (early) feed was contaminated with the highest levels of DON out of the swine feed samples. Eighty-six percent of the poultry feed samples were contaminated with DON, and the levels of contamination ranged from 0.035 to 1.492 mg/kg, which was lower than that listed by commission regulation of the European Community. Consistent with these observations, Labuda *et al.* (2005) reported that 56% of poultry feed samples were contaminated with DON, and that the levels of contamination ranged from 0.064 to 1.230 mg/kg, with a mean of 0.303 mg/kg [10].

**Table 1:** The presence of deoxynivalenol (DON) in compound feeds and feed ingredients.

Samples				Number (%) of DON contaminated samples	Concentration (mg/kg)		
Type		Total number	Contaminated number		Minimum ± SD	Maximum ± SD	Mean ± SD
**Cattle feeds**							
Breeding	Pregnancy	5	5	(100^b^)	0.225 ± 0.030	0.869 ± 0.004	0.463 ± 0.248
Lactation	Lactation	11	11	(100^b^)	0.131 ± 0.027	1.000 ± 0.084	0.466 ± 0.280
	Calf	11	11	(100^b^)	0.137 ± 0.018	0.942 ± 0.066	0.465 ± 0.280
Fatting	Calves	11	11	(100^b^)	0.182 ± 0.002	0.943 ± 0.148	0.585 ± 0.282
	Finishing (early^c^)	6	6	(100^b^)	0.213 ± 0.008	0.818 ± 0.181	0.470 ± 0.256
	Finishing (late^d^)	6	6	(100^b^)	0.187 ± 0.036	0.885 ± 0.030	0.642 ± 0.334
Total		50	50	(100^b^)			
**Swine feeds**							
	Piglets	5	2	(40^b^)	0.046 ± 0.002	0.116 ± 0.020	0.081 ± 0.049
	Piglets ≥ 5 kg	10	10	(100^b^)	0.114 ± 0.011	0.781 ± 0.038	0.325 ± 0.223
Breeding	Pregnancy	10	10	(100^b^)	0.097 ± 0.005	0.732 ± 0.011	0.356 ± 0.253
	Lactation	10	8	(80^b^)	0.038 ± 0.006	0.843 ± 0.057	0.308 ± 0.315
Fatting	Growing (early^c^)	10	9	(90^b^)	0.037 ± 0.024	0.982 ± 0.329	0.321 ± 5.032
	Growing (late^d^)	5	5	(100^b^)	0.043 ± 0.008	0.492 ± 0.014	0.151 ± 0.193
Total		50	44	(88 ^b^)			
**Poultry feeds**							
Layer	Chicken	15	15	(100^b^)	0.045 ± 0.001	1.097 ± 0.201	0.282 ± 0.297
	Laying	11	10	(91^b^)	0.035 ± 0.008	1.492 ± 0.448	0.255 ± 0.452
Broiler	Broiler (early^c^)	10	8	(80^b^)	0.042 ± 0.021	0.139 ± 0.023	0.089 ± 0.038
	Broiler (late^d^)	9	7	(78^b^)	0.051 ± 0.026	0.333 ± 0.009	0.145 ± 0.096
Parent stock	Breeder	5	3	(60^b^)	0.079 ± 0.013	0.749 ± 0.181	0.329 ± 0.336
Total		50	43	(86^b^)			
**Feed ingredients**							
Vegetable proteins	Soybean meal	2	0	(0)		-	-
	Corn gluten	4	1	(25^b^)	0.050 ± 0.015	0.050 ± 0.015	0.050 ± 0.015
	Corn germ meal	2	2	(100^b^)	0.083 ± 0.005	0.094 ± 0.004	0.089 ± 0.007
	Others^e^	10	6	(60^b^)	0.555 ± 0.031	0.957 ± 0.003	0.663 ± 0.150
Bran	Wheat bran	1	0	(0)	-	-	-
	Others^f^	8	7	(87.5^b^)	0.075 ± 0.012	0.640 ± 0.099	0.421 ± 0.232
Others	By-products of food	2	0	(0^b^)	-	-	-
	Grain products	1	0	(0)	-	-	-
Total		30	16	(53.33^b^)			
Total		180	153	(85 ^b^)			

Notes: ^a^: Samples with DON concentrations ≥LOQ (0.035 mg/kg) were used in the analyses; ^b^: Concentration (%); ^c^: Early: under three months; ^d^: Late: 3 to 6 months; ^e^: Others: distillers dried grains, palm oil meal, coffee meal; ^f^: Others: corn gluten feed, wheat bran, cotton seeds hull, corn-bran, wheat shorts, wheat flour.

Table 1 also explained DON contamination and 53.33% of the feed ingredient samples were contaminated with DON, with a mean concentration of 0.447 mg/kg, ranging from 0.050 to 0.957 mg/kg. These levels were lower than those listed by the commission regulation of the European Community. The highest level of DON was observed in a vegetable protein samples (0.957 mg/kg) among all the feed ingredients tested. The contamination range of DON in feed ingredient samples was consistent with the observations of Binder *et al.* (2007), who analyzed feeds and feed ingredients for the presence of DON in North Asia [11]. Unlike aflatoxin B_1_, DON was highly prevalent in that region (71%), and the highest level (18.991 mg/kg) was detected in a wheat sample sourced in China. In their study, the mean level of DON was 0.162 mg/kg and the level was lower than our study. 

Consistent with the results in the present study, Monbaliu *et al*. (2010) reported that DON was detected in 52 samples with mean level of 0.949 mg/kg and 12 samples were contaminated with ZEN as mean level of 0.157 mg/kg. The samples were collected from three EU countries; Czech Republic, Denmark, and Hungary. This report also presented the contamination of FB_1_ and FB_2_ in 36 and 29 samples with mean levels of 0.913 and 0.292 mg/kg, respectively [12]. Several research articles reported that the levels of mycotoxins in compound feeds were lower than those observations in feed ingredients [13,14] mainly due to the dilution of mycotoxins by cleaning and mixing steps during processing. However, in our previous reports related to contamination of *Fusarium* mycotoxin (FB_1_, FB_2_, and beauvericin) in animal feeds in Korea, we also found higher contamination levels in compound feeds than those levels in feed ingredient [15,16] as shown in the present study. This tendency can be explained by following two points of view. First, the contamination levels of *Fusarium* mycotoxins in animal feeds and feed ingredients in Korea are relatively lower than those observations in other countries or much the same [4,10,11,18]. Therefore, the tendency that the levels of contamination observed in compound feeds are higher than those reported for feed ingredient can be observed mainly due to the relatively lower contamination levels of *Fusarium* mycotoxins in feed ingredients. Second, several compound feeds in Korea contain corn-gluten feed (between 10% and 20%) as a feed ingredient which showed the highest levels of *Fusarium* mycotoxin, as shown in “others” presented in Table 1, Table 2 and Table 3. The addition of this feed ingredient could be the main reason why the levels of contamination observed in compound feeds are high compared with those levels in feed ingredients. Further study should be performed to explain this observation by investigation of storage conditions, processing steps and distribution of compound feeds in Korea.

#### Contamination Levels of FBs in Compound Feeds and Feed Ingredients

As shown in Table 2, FBs were detected in 98% of cattle feeds, at concentrations ranging from 0.042 to 2.990 mg/kg. The lactation calf feed exhibited the highest contamination of FB_S_, with a mean contamination level of 0.691 mg/kg. Eighty-two percent of the 50 tested samples of swine feed were contaminated with FBs, with levels ranging from 0.037 to 5.509 mg/kg. The breeding pregnancy feed was contaminated with the highest levels of FBs, and the mean level of contamination was 1.108 mg/kg. These FBs levels were lower than the levels previously reported by our research group in animal feeds distributed in South Korea in 2011 [16] and they were all lower than those mentioned in the commission regulation of the European Community. However, Martins *et al.* (2011) reported that 8.7% of swine feed samples in Portugal were contaminated with FBs in 2010, which is significantly lower than the percentage observed in this study [17]. All of the poultry feed samples were contaminated with FBs, and the contamination levels ranged from 0.092 to 12.823 mg/kg, with a mean of 2.181 mg/kg. Broiler (early) feeds contained the highest contamination with FBs, with mean levels of 3.459 mg/kg. A similar contamination level of FBs was reported by Klarić *et al.* (2009) in Croatia with a mean level of 3.690 mg/kg in cereals and contaminated feed collected in 2007 from households of an endemic nephropathy area [18].

**Table 2:** The presence of fumonisins B1 and B2 (FBs) in compound feeds and feed ingredient samples.

Samples				Number (%) of FBs contaminated samples	Concentration (mg/kg)		
Type		Total number	Contaminated number		Minimum ± SD	Maximum ± SD	Mean ± SD
**Cattle feeds**							
Breeding	Pregnancy	5	4	(80^b^)	0.102 ± 0.033	1.125 ± 0.000	0.492 ± 0.449
Lactation	Lactation	11	11	(100^b^)	0.042 ± 0.003	2.560 ± 2.508	1.038 ± 1.000
	Calf	11	11	(100^b^)	0.045 ± 0.005	2.990 ± 0.000	0.691 ± 0.851
Fatting	Calves	11	11	(100^b^)	0.099 ± 0.003	2.007 ± 0.154	0.803 ± 0.637
	Finishing (early^c^)	6	6	(100^b^)	0.556 ± 0.049	1.472 ± 0.117	1.130 ± 0.355
	Finishing (late^d^)	6	6	(100^b^)	0.082 ± 0.000	2.020 ± 0.102	0.584 ± 0.757
Total		50	49	(98^b^)			
**Swine feeds**							
	Piglets	5	5	(100^b^)	0.038 ± 0.005	0.420 ± 0.028	0.255 ± 0.139
	Piglets ≥ 5 kg	10	7	(70^b^)	0.037 ± 0.000	1.160 ± 0.002	0.387 ± 0.388
Breeding	Pregnancy	10	8	(80^b^)	0.120 ± 0.000	5.509 ± 0.008	1.108 ± 1.847
	Lactation	10	9	(90^b^)	0.065 ± 0.000	0.680 ± 0.122	0.290 ± 0.332
Fatting	Growing (early^c^)	10	7	(70^b^)	0.114 ± 0.025	1.280 ± 0.113	0.359 ± 0.421
	Growing (late^d^)	5	5	(100^b^)	0.097 ± 0.009	0.257 ± 0.082	0.183 ± 0.064
Total		50	41	(82^b^)			
**Poultry feeds**							
Layer	Chicken	15	15	(100^b^)	0.145 ± 0.008	9.383 ± 0.013	1.577 ± 2.384
	Laying	11	11	(100^b^)	0.315 ± 0.058	4.530 ± 0.093	2.314 ± 1.818
Broiler	Broiler (early^c^)	10	10	(100^b^)	0.333 ± 0.006	12.823 ± 4.442	3.459 ± 3.840
	Broiler (late^d^)	9	9	(100^b^)	0.092 ± 0.035	4.757 ± 5.247	1.614 ± 1.488
Parent stock	Breeder	5	5	(100^b^)	0.299 ± 0.000	4.382 ± 0.024	2.166 ± 1.502
Total		50	50	(100^b^)			
**Feed ingredients**							
Vegetable proteins	Soybean meal	2	2	(100^b^)	0.059 ± 0.003	0.150 ± 0.008	0.105 ± 0.064
	Corn gluten	3	3	(100^b^)	0.805 ± 0.029	2.694 ± 0.079	1.477 ± 1.056
	Corn germ meal	2	2	(100^b^)	0.283 ± 0.004	0.534 ± 0.005	0.409 ± 0.177
	Others^e^	11	7	(54.55^b^)	0.216 ± 0.001	2.748 ± 0.030	1.539 ± 1.137
Bran	Wheat bran	1	1	(100^b^)	0.069 ± 0.000	0.069 ± 0.000	0.069 ± 0.000
	Others^f^	7	7	(100^b^)	0.565 ± 0.041	8.239 ± 0.116	3.378 ± 3.241
Others	By-products of food	3	3	(100^b^)	0.066 ± 0.000	0.164 ± 0.006	0.106 ± 0.052
	Grain products	1	0	(0)	-	-	-
Total		30	25	(83.33^b^)			
Total		180	165	(91.67^b^)			

Notes: ^a^: Samples with FB_1_ concentrations ≥LOQ (0.030 mg/kg) and FB_2_ concentrations ≥LOQ (0.035 mg/kg) were used for analysis; ^b^: Concentration(%); ^c^: Early: under 3 months; ^d^: Late: 3 to 6 months; ^e^: Others: distillers dried grains, palm oil meal, coffee meal; ^f^: Others: corn gluten feed, wheat bran, cotton seeds hull, corn-bran, wheat shorts, wheat flour.

Several research articles reported contamination of FBs in forms of FB_1_ and FB_2,_ separately. In a study of simultaneous occurrence of FB_1_ in China, Chu and Li (1994) reported that the corn collected from two regions was heavily contaminated with FB_1_ at a mean level of 94 mg/kg [4]. In the investigation of Portuguese corn-based products, Waskiewicz *et al.* (2012) reported that FB_1_ was detected in all corn flour samples analyzed, and FB_2_ was detected in 70.7% of tested samples. The concentrations of FB_1_ and FB_2_ ranged from 0.050 to 1.300 mg/kg, and 0.100–0.450 mg/kg, respectively [19]. Consistent with other studies, the contamination levels of FB_2_ were lower than those of FB_1_ in all tested animal feeds and feed ingredients and all FB_2_ contaminated samples were co-contaminated with FB_1_ [20,21].

Table 2 also presented contamination of FBs in 25 feed ingredient samples (83.33%), with lower contamination levels ranging from 0.059 to 8.239 mg/kg, and a mean contamination level of 1.611 mg/kg compare with those observations in compound feeds. These levels were lower than the levels of FBs described by the commission regulation of the European Community. The data in these results were consistent with those reported by Binder *et al.* (2007), who assessed the presence of FBs (FB_1_, FB_2_, and FB_3_) in feed ingredients in Asia and Oceania [11]. The highest level of contamination with FBs was found in a feed ingredient from China, with concentrations of 14.714 mg/kg. The total contamination incidence in China was 16% in feed ingredients that were collected from the same regions.

#### Contamination Levels of ZEN in Compound Feeds and Feed Ingredients

ZEN was detected in 98% of the cattle feeds, with concentrations ranging from 0.009 to 0.405 mg/kg (Table 3). The fatting calves feed exhibited the highest contamination of ZEN, with a mean contamination of 0.194 mg/kg. In the study of cattle feeds in Turkey, Kocasari *et al.* (2013) reported that ZEN was detected in 45.2% of tested samples at concentrations ranging from 0.002 to 0.029 mg/kg [21]. Sixty percent of the 50 swine feed samples were contaminated with ZEN, at concentrations ranging from 0.008 to 0.207 mg/kg at a mean of 0.036 mg/kg. Out of the swine feed samples, breeding lactation feed was contaminated with the highest levels of ZEN. A total of 56% of the poultry feed samples were contaminated with ZEN, and the contamination levels ranged from 0.008 to 0.228 mg/kg, with a mean of 0.041 mg/kg. This level is lower than that listed in the commission regulation of the European Community. Consistent with our observations, Labuda *et al.* (2005) reported that 88% of poultry feed samples were contaminated with ZEN, and that levels ranged from 0.003 to 0.086 mg/kg (mean 0.021 mg/kg) in Slovakia in 2004 [10].

Forty-seven percent of feed ingredients were contaminated with ZEN (14 feed ingredient samples out of 30 samples) at the concentrations range of 0.010–0.413 mg/kg. The mean contamination level was 0.123 mg/kg, which is lower than the levels of ZEN specified in the commission regulation of the European Community. Interestingly, all tested corn gluten and corn germ meal samples were contaminated with ZEN. Several researchers in different countries have reported the presence of ZEN in feed ingredients. Binder *et al.* (2007) analyzed the presence of ZEN in feed ingredients in Asia and Oceania [11], and reported mean ZEN contamination levels of 0.077 mg/kg in feed ingredients, which is comparable to our observations. The total incidence of contamination was 24% in feed ingredients that were collected from the same regions, which is much lower than those observed in this study.

**Table 3:** The presence of zearalenone (ZEN) in compound feeds and feed ingredient samples.

Samples				Number (%) of ZEN contaminated samples	Concentration (mg/kg)		
Type		Total number	Contaminated number		Minimum ± SD	Maximum ± SD	Mean ± SD
**Cattle feeds**							
Breeding	Pregnancy	5	5	(100^b^)	0.011 ± 0.001	0.100 ± 0.050	0.057 ± 0.038
Lactation	Lactation	11	11	(100^b^)	0.010 ± 0.004	0.262 ± 0.117	0.095 ± 0.101
	Calf	11	10	(91^b^)	0.009 ± 0.000	0.265 ± 0.008	0.094 ± 0.024
Fatting	Calves	11	11	(100^b^)	0.029 ± 0.016	0.405 ± 0.087	0.194 ± 0.134
	Finishing (early^c^)	6	6	(100^b^)	0.024 ± 0.008	0.153 ± 0.038	0.073 ± 0.058
	Finishing (late^d^)	6	6	(100^b^)	0.015 ± 0.004	0.186 ± 0.005	0.114 ± 0.069
Total		50	49	(98^b^)			
**Swine feeds**							
	Piglets	5	1	(20^b^)	0.200 ± 0.257	0.200 ± 0.257	0.200 ± 0.257
	Piglets ≥ 5 kg	10	6	(60^b^)	0.008 ± 0.001	0.046 ± 0.003	0.029 ± 0.019
Breeding	Pregnancy	10	6	(60^b^)	0.017 ± 0.004	0.086 ± 0.013	0.032 ± 0.017
	Lactation	10	6	(60^b^)	0.008 ± 0.004	0.207 ± 0.048	0.046 ± 0.019
Fatting	Growing (early^c^)	10	7	(70^b^)	0.008 ± 0.005	0.055 ± 0.047	0.026 ± 0.020
	Growing (late^d^)	5	4	(80^b^)	0.009 ± 0.002	0.019 ± 0.027	0.013 ± 0.002
Total		50	30	(60^b^)			
**Poultry feeds**							
Layer	Chicken	15	10	(67^b^)	0.010 ± 0.000	0.132 ± 0.006	0.044 ± 0.012
	Laying	11	3	(27^b^)	0.013 ± 0.011	0.228 ± 0.008	0.109 ± 0.012
Broiler	Broiler (early^c^)	10	5	(50^b^)	0.008 ± 0.001	0.020±0.006	0.012 ± 0.006
	Broiler (late^d^)	9	8	(89^b^)	0.009 ± 0.002	0.038 ± 0.003	0.027 ± 0.009
Parent stock	Breeder	5	2	(40^b^)	0.012 ± 0.002	0.087 ± 0.020	0.050 ± 0.011
Total		50	28	(56^b^)			
**Feed ingredients**							
Vegetable proteins	Soybean meal	2	0	(0^b^)	-	-	-
	Corn gluten	4	1	(25^b^)	0.148 ± 0.001	0.148±0.001	0.148 ± 0.001
	Corn germ meal	2	2	(100^b^)	0.014 ± 0.002	0.413 ± 0.004	0.327 ± 0.087
	Others^e^	10	5	(50^b^)	0.013 ± 0.003	0.077 ± 0.096	0.029 ± 0.023
Bran	Wheat bran	1	0	(0)	-	-	-
	Others^f^	8	6	(75^b^)	0.010 ± 0.004	0.291 ± 0.004	0.129 ± 0.028
Others	By-products of food	1	0	(0)			
	Grain products	2	0	(0)	-	-	-
Total	30	14	(47^b^)				
Total	180	121	(67.22^b^)				

Notes: ^a^: Samples with ZEN concentrations ≥LOQ (0.008 mg/kg) were used for analysis; ^b^: Concentration (%); ^c^: Early: under three months; ^d^: Late: 3 to 6 months; ^e^: Others: distillers dried grains, palm oil meal, coffee meal; ^f^: Others: corn gluten feed, wheat bran, cotton seeds hull, corn-bran, wheat shorts, wheat flour.

#### Co-contamination of the Three Toxins in Compound Feeds and Feed Ingredients

Co-contamination of *Fusarium* mycotoxins is of great of interest as mycotoxigenic fungi are capable of producing more than one mycotoxin and feed raw materials might be infected with various fungal species. Thus, studying the occurrence of any given mycotoxin alone does not provide sufficient information about the risk associated with the respective feedstuffs [22]. Simultaneous occurrence of mycotoxins appears to exert greater negative effects on health and productivity than single mycotoxins [23], if there is no appropriate investigation, and management of mycotoxins in feeds and all relevant precautions have to be considered.

As illustrated in Table 4, most of the samples showed co-occurrence of mycotoxins at the same time. Cattle feeds showed the highest percentage of co-occurrence of mycotoxins followed by poultry feeds, swine feeds and ingredient samples. Some samples heavily contaminated with FBs or DON and usually contaminated with ZEN weakly. For example, one broiler (early) feed for poultry exhibited the maximum contamination level of FBs (12.823 mg/kg) were co-contaminated with the lower levels of DON (0.139 mg/kg) and ZEN (0.020 mg/kg). Other poultry feed for layer (laying) with the highest contamination level of DON (1.492 mg/kg) was also co-contaminated with FBs (4.530 mg/kg) and ZEN (0.228 mg/kg), respectively.

**Table 4:** Co-contamination of three toxins in animal feeds.

Categories	DON + FBs + ZEN	The range of DON (mg/kg)	The range of FBs (mg/kg)	The range of ZEN (mg/kg)
Cattle feeds	48/50	0.131–1.000	0.042–2.990	0.009–0.405
Swine feeds	25/50	0.037–0.982	0.037–5.509	0.008–0.207
Poultry feeds	27/50	0.035–1.492	0.092–12.823	0.008–0.228
Feed ingredient	12/30	0.050–0.957	0.059–8.239	0.010–0.413
Total	112/180			

## 3. Materials and Methods

### 3.1. Samples

Compound feed and feed ingredient samples were collected from various regions in South Korea, and provided by the National Agriculture Products Quality Management Service Experiment Research Institute in 2012. The samples were prepared following the sampling guide in the code for the control of feeds (FAO/WHO 2004). One kilogram of sample was taken randomly from every ton of feed ingredients or compound feeds. Four different samples were pooled and divided into four groups, according to the sampling guide in the code for the control of feeds. Five hundred grams of the divided samples were tested for contamination with DON, FB_1_, FB_2,_ and ZEN. The concentrations of DON, FB_1_, FB_2,_ and ZEN were assessed in 150 samples of compound feeds, including cattle feed, swine feed, and poultry feed, and 30 samples of feed ingredients including vegetable proteins, and bran. The samples were stored at 4 °C before analysis to maintain the condition of the samples.

### 3.2. Extraction and Purification of DON, FB_S_, and ZEN

Feed samples were milled to obtain a particle size of 600 μM. DON was analyzed and validated as described in the Korean Food Standards Codex method (2010) for the determination of DON in corn and corn flakes. Twenty grams of each feed sample was extracted in 100 mL distilled water by homogenizing (OMNI MACRO ES HOMOGENIZER, USA) for 2 min at 10,000 rpm. The extract was then filtered through Whatman No. 6 filter paper, and 2.5 mL of filtered extract was applied to the immunoaffinity column (IAC; Vicam, DON test) containing specific antibodies for DON for purification. The IAC was washed with 5 mL 100% water, and DON was slowly eluted in 3 mL 100% methanol at 1 drop/s. The elute was collected and evaporated to dryness under nitrogen steam (50 °C).

The analysis and validation of FB_1_ and FB_2_ were carried out in all samples according to the AOAC official method for the determination of fumonisins B_1_ and B_2_ (AOAC 2001). Twenty grams of feed sample was extracted with 100 mL of extraction solvent (25:25:50 methanol:acetonitrile:water, v/v/v) using a homogenizer for 2 min at 10,000 rpm. The extract was then filtered through Whatman No. 6 filter paper. Ten milliliters of the filtered extract was diluted in 40 mL phosphate-buffered saline (PBS; Sigma-Aldrich, St. Louis, MO, USA), and the diluted extracts were collected and purified through an IAC-containing fumonisin-specific antibodies. The IAC was rinsed with 10 mL PBS, and FB_1_ and FB_2_ were then eluted from the column using 4 mL 80% methanol at 1 drop/s. The eluted fraction was evaporated to dryness under nitrogen steam at 60 °C, and stored at 4 °C for derivatization before being analyzed by high-performance liquid chromatography (HPLC).

The ZEN assay and validation were performed as the Korean Food Standards Codex method (2010) for quantifying ZEN in corn and corn flakes. Twenty-five grams of each feed sample was extracted with 100 mL 75% acetonitrile containing 1 mL Tween20 and 2 g NaCl at 10,000 rpm for 5 min. The extract was filtered through Whatman No.4 filter paper, and 10 mL filtered extract was diluted with 40 mL distilled water. Twenty-five milliliters of diluted extract was then purified using an IAC. The IAC was washed with 20 mL water at a flow-rate of 1 drop per second, and the elute was collected in 5 mL methanol and evaporated using nitrogen steam (40 °C).

### 3.3. HPLC Analysis

For DON, the dried samples were re-dissolved in 0.5 mL of acetonitrile:water (17:83, v/v). The samples (20 μL) were analyzed using HPLC (Agilent Technology 1260 series; Agilent, Santa Rosa, CA, USA) equipped with an Agilent Technology 1200 Infinity UV Detector. An analytical column (ZORBAX Eclipse XDB-C18 column, 4.6 × 250 mm, 5 μM, Agilent, USA) was used for HPLC analysis for 20 min at a constant flow rate of 1 mL/min at 30 °C, and DON was detected at an absorbance of 220 nm.

To analyze FB_1_ and FB_2_, the dried samples from IAC were re-dissolved in 0.5 mL acetonitrile:water (50:50, v/v). Because this sample did not exhibit any fluorescence, it was derivatized using *o*-phthalaldehyde (OPA) reagent. Twelve microliters OPA was mixed with 10 μL sample using an auto-program in the HPLC (Agilent Technology 1200 series; Agilent, USA) equipped with Agilent Technology 1260 Infinity Fluorescence Detector. The isocratic mobile phase (77:23 MeOH:0.1 M NaH_2_PO_4_, adjusted to pH 3.3 with H_3_PO_4_) and analytical column (ZORBAX Eclipse XDB-C18 column, 4.6 × 250 mm, 5 μM, Agilent, USA) were used for HPLC analysis for 30 min at a constant flow rate of 1 mL/min at 30 °C. FB_1_ and FB_2_ were detected by fluorescence with an excitation at 335 nm and emission at 440 nm.

For ZEN, HPLC analysis was performed on an Agilent Technology 1200 series machine (Agilent, USA) for 20 min at a flow-rate of 1 mL/min. The mobile phase consisted of a mixture of methanol-water-acetonitrile (55:35:10, v/v/v), and an analytical column (ZORBAX Eclipse XDB-C18 column, 4.6 × 250 mm, 5 mm, Agilent, USA) was used for HPLC analysis. Twenty milliliters reconstituted extract was injected into the HPLC, and ZEN was measured (Agilent Technology 1260 Infinity Fluorescence Detector; Agilent, USA) with an excitation at 275 nm and emission at 450 nm.

The analysis was done in duplication and the data was presented with ± SD which indicated mean ± SD of replication done on different samples of the same feed.

### 3.4. Limit of Detection (LOD) and Limit of Quantification (LOQ)

DON standard powder was purchased from Sigma-Aldrich (St. Louis, Mo, USA), and a standard solution of DON (1 mg/mL) in 17% acetonitrile was stored in a sealed vial at −20 °C until use. A standard solution of mixed FB_1_ and FB_2_ containing 50 μg/mL each mycotoxin was purchased from Sigma-Aldrich, and diluted in 50% acetonitrile to create 0.5 μg/mL standard solution, which was stored until needed to make a standard curve. ZEN standard powder was also purchased from Sigma Aldrich and, 1 mg powder was dissolved in 100% acetonitrile as the stock solution, which was then further diluted in 100% acetonitrile to make the standard curve. The LOD was calculated at three times the signal-to-noise ratio, and LOQ was calculated at 10 times the signal-to-noise ratio.

The limit of detection (LOD) of DON was 0.010 mg/kg, and the limit of quantification (LOQ) was 0.035 mg/kg. Consistent with this, Bensassia *et al.* (2010) reported an LOD of 0.010 mg/kg and an LOQ of 0.030 mg/kg [24].

For FB_1_ and FB_2_, the LODs were 0.020 and 0.025 mg/kg, respectively, and the LOQs were 0.030 mg/kg for FB_1_ and 0.035 mg/kg for FB_2_. In a previous study, Krska *et al.* (2007) reported that the LOD of FBs in feeds ranged from 0.010 to 0.050 mg/kg sample dry weights, which is consistent with our observations [25]. However, they reported LOQs of FBs in feeds in the range of 0.070–0.090 mg/kg [10], which is significantly higher than those reported from the present study.

For ZEN, the LOD and LOQ were 0.003 mg/kg and 0.008 mg/kg, respectively. Previously, Eskola *et al.* (2002) reported an LOD of ZEN of 0.002 mg/kg, and an LOQ of 0.003 mg/kg [26], which is lower than the LOD detected in our study.

### 3.5. LC-MS/MS Analysis

Liquid chromatography-tandem mass spectrometry (LC-MS/MS; Agilent HPLC, ABI 4000, USA) was used to confirm the type of DON and ZEN in the negative electrospray ionization (ESI−) mode, and FB_1_ and FB_2_ in the positive mode (ESI+). An analytical column (Imtakt Scherzo SM-C18) was used (2.0 × 150 mm, 3 μM) under gradient conditions with a flow rate of 0.25 mL/min with the mobile phase consisting of 20% acetonitrile containing 2 mM ammonium acetate, and acetonitrile containing 0.3% formic acid. Ten microliters sample was injected in to the HPLC and LC-MS/MS system, and the molecular weights of analyzed mycotoxins were compared with of the standard solutions. The source voltage was set in as 4.5 kV at positive mode and −4.5 kV at negative mode with ion source temperature at 450 °C. In the negative mode, the precursor ion [M + CH_3_COOH]^−^ of DON exhibited an m/z value of 355.1, with daughter product ions at 295.2 and 58.9 in MS/MS analysis. In the positive mode, the precursor ion [M + H]^+^ of FB_1_ showed m/z of 722.4, which resulted in daughter product ions of 336.3 and 334.3 in MS/MS analysis. The EIC and MRM of FB_2_, in which the peak of precursor ion [M + H]^+^ was found at an m/z value of 706.4, with daughter product ions at 336.3 and 273.1 in the same mode of MS/MS analysis. ZEN in samples was detected as a peak of m/z 317, which was consistent with the M/Z value of the ZEN standard. The M/Z values of the daughter product ions were 131.0 and 175.0.

## 4. Conclusions

Among the tested compound feeds and feed ingredients, 91.67% of the animal feeds were contaminated with FBs. In addition, 85% were contaminated with DON, and 67.22% were contaminated with ZEN. The contamination levels of DON, FBs, and ZEN were 0.035–1.492, 0.037–12.823, and 0.008–0.413 mg/kg, respectively. This study revealed that the contamination levels of *Fusarium* mycotoxins (DON, FBs, and ZEN) were lower than the maximum levels set by the commission regulation of the European Community (EU 2006) except one sample for swine feed (FBs, 5.5 mg/kg). However, the percentage of mycotoxin samples was noticeably high (67%–92%), suggesting that most animal feeds distributed in South Korea are contaminated. In addition, co-contamination of *Fusarium* mycotoxins in most samples makes it more concerning for animal health and reproduction and all relevant precautions have to be considered. Moreover, guidelines or maximum levels should not only be set for each mycotoxin individually but also for particularly concerning combinations of these toxins as it would be a more effective way to monitor feed safety in relation to mycotoxin contamination, and continuous monitoring of mycotoxins in feeds is therefore necessary for improving feed safety.
